# Structural brain changes in subacute spinal cord injury: an analysis of diffusion kurtosis imaging and diffusion tensor imaging metrics with clinical correlation

**DOI:** 10.3389/fnins.2025.1652416

**Published:** 2025-11-28

**Authors:** Ernst Christiaanse, Jothini Sritharan, Patrik O. Wyss, Anke Scheel-Sailer, Alexander Leemans, Rajeev K. Verma, Alberto De Luca, Giuseppe A. Zito

**Affiliations:** 1 Department of Radiology, Swiss Paraplegic Centre, Nottwil, Switzerland; 2 Image Sciences Institute, Center for Image Sciences, University Medical Center Utrecht, Utrecht, Netherlands; 3 Swiss Paraplegic Research, Nottwil, Switzerland; 4 Faculty of Health Sciences and Medicine, University Lucerne, Lucerne, Switzerland; 5 Centre for Rehabilitation and Sports Medicine, Inselspital and Bern Rehab Centre, Bern University Hospital, University Bern, Bern, Switzerland; 6 Department of Neurology, UMC Utrecht Brain Center, University Medical Center Utrecht, Utrecht, Netherlands

**Keywords:** structural brain changes, spinal cord injury, diffusion kurtosis imaging (DKI), diffusion tensor imaging (DTI), clinical correlation

## Abstract

**Introduction:**

Diffusion tensor imaging (DTI) and diffusion kurtosis imaging (DKI) can quantify indices related to brain structure and their change in pathology. However, only few studies have applied these techniques to spinal cord injury (SCI), and subtle microstructural changes in the brain of SCI individuals are not well understood. Our goal was to investigate structural changes in the brain using DTI (fractional anisotropy, FA; mean diffusivity, MD) and DKI parameters (kurtosis anisotropy, KA; mean kurtosis, MK) in subacute SCI and to study whether these changes were associated with clinical outcomes.

**Methods:**

Twenty-eight individuals with SCI underwent brain MRI 3 months post-injury, alongside 20 healthy controls. Imaging included a multi-shell diffusion protocol, from which DTI and DKI metrics (FA, MD, KA and MK) were derived. Group comparisons were conducted for each metric across 17 brain regions selected based on their relevance to SCI from previous studies. Multiple comparison corrections were applied per metric to account for the number of examined regions. Effect sizes were calculated using Cohen’s *d*. For regions showing significant group differences, Spearman correlations were performed to assess associations between imaging metrics and clinical outcomes, including neurological status (ISNCSCI) and functional independence (SCIM III), with correction for multiple comparisons.

**Results:**

MD was significantly higher in the right genu of the corpus callosum in the SCI group (adjusted *p* = 0.021). In this region, MD negatively correlated with SCIM scores (*r* = −0.51, *p* = 0.022), whereas MK showed a positive correlation (*r* = 0.482, *p* = 0.038).

**Discussion:**

Structural changes in the corpus callosum may reflect impaired interhemispheric communication, linked to reduced functional independence after SCI. DTI and DKI could serve as complementary tools for identifying brain-based biomarkers, potentially informing recovery trajectories.

## 1 Introduction

Spinal cord injury (SCI) is a serious condition that significantly affects quality of life. In the United States, individuals with SCI have a mortality rate three times higher than those without SCI (DeVivo et al., 2022). In 2021, approximately 15.4 million people were living with SCI worldwide (WHO, 2024). Although advances in the treatment and management of SCI have been made, the effects of SCI on the reorganization of the brain and of the spinal cord remain not well understood. Mapping such changes thus represents an important step to better understand SCI and could aid the development of targeted treatments and rehabilitation strategies.

Previous studies (Freund et al., 2013; Freund et al., 2011; Bammer, 2003) have shown that SCI does not only result in at-level and below-level neurological impairment, but also in changes of the spinal cord above the injury and in the brain. For instance, Freund et al. demonstrated extensive upstream atrophic and microstructural changes of corticospinal axons and sensorimotor cortical areas occurring within the first months after SCI, with faster degenerative changes linked to poorer recovery (Freund et al., 2013; Freund et al., 2011). These changes can be identified using various imaging modalities, including T1-weighted and diffusion-weighted imaging (DWI). Notably, DWI is a promising method for detecting alterations in white matter structures (Bammer, 2003). It measures the random microscopic movement (Brownian motion) of water molecules within a tissue and, in the last decades, has been used for routine clinical applications. An established technique based on DWI is diffusion tensor imaging (DTI). DTI has proven to be sensitive to microstructural changes in several neurological conditions, and has been successfully applied in amyotrophic lateral sclerosis (Zhang et al., 2018; Foerster et al., 2013), multiple sclerosis (Song et al., 2002; Rovaris et al., 2005), Parkinson’s disease (Cochrane and Ebmeier, 2013) and Alzheimer’s dementia (Mayo et al., 2017). Yet, only a few studies in the field of SCI have applied DTI to investigate microstructural brain changes and their clinical correlations. For instance, Guo et al. demonstrated that SCI individuals had decreased fractional anisotropy (FA) and increased mean diffusivity (MD) and radial diffusivity (RD) in the corpus callosum (genu and splenium), superior longitudinal fasciculus, corona radiata, posterior thalamic radiation, right cingulate gyrus and right superior fronto-occipital fasciculus (Guo et al., 2019). Additionally, time since injury was negatively correlated with FA in the right superior fronto-occipital fasciculus, while FA in the left posterior thalamic radiation was positively associated with the American spinal injury association (ASIA) sensory scores. Zheng et al. demonstrated that, compared with non-injured controls, SCI individuals exhibited significant decreases in white matter FA in the left angular gyrus, right cerebellum, left precentral gyrus, left lateral occipital region, left superior longitudinal fasciculus, left supramarginal gyrus, and left postcentral gyrus (Zheng et al., 2017). Furthermore, a significant negative correlation was observed between increased RD in the left angular gyrus and total motor scores. These changes have been linked to demyelination and abnormal sensory perception, as well as motor impairment (Guo et al., 2019). Huynh et al. reported reduced FA in the thalamic radiation and corticospinal tract following SCI, with motor function also showing associations with both FA and MD of the corticospinal tract (Huynh et al., 2021). Sun et al. demonstrated significantly lower FA in the cerebral peduncles in individuals with cervical SCI compared to healthy controls (Sun et al., 2017). Finally, Ilvesmäki et al. reported significant white matter changes across the cerebrum, affecting projection (including the corticospinal tract and thalamocortical tracts), commissural (notably the genu and anterior body of the corpus callosum), and association fibers (including the inferior/superior longitudinal fasciculi, inferior fronto-occipital fasciculus, uncinate fasciculus, and anterior cingulum), with CST alterations extending from the cerebral peduncle to subcortical motor and sensory areas (Ilvesmaki et al., 2017).

These widespread brain abnormalities highlight the need for more advanced diffusion imaging approaches, which have become increasingly feasible with recent advancements in MRI scanner technology and multi-shell acquisition protocols. To leverage the information content of such acquisitions, other diffusion MRI techniques beyond DTI have been introduced, such as diffusion kurtosis imaging (DKI) (Jensen et al., 2005; Jensen and Helpern, 2010). DKI quantifies the deviation of the diffusion process from Gaussianity due to the effect of biological membranes, among others (Kärger, 1985). Several studies (Guglielmetti et al., 2016; Arab et al., 2018) have demonstrated that DKI has an increased sensitivity to pathological changes, as compared to DTI. For instance, microstructural changes, such as cortical demyelination, white and gray matter changes, typically observed in multiple sclerosis, have been studied with DKI in mouse models (Guglielmetti et al., 2016). DKI was also able to detect early microstructural changes in neurodegenerative disorders such as Alzheimer’s disease and Parkinson’s disease (Arab et al., 2018). Furthermore, a previous study demonstrated that DTI-derived metrics based on the kurtosis framework are more accurate compared to DTI (Henriques et al., 2021). However, DKI has so far only been applied in a single study investigating changes in the cervical spinal cord following spinal cord injury (Thygesen et al., 2022).

The previously referenced studies on DTI in SCI were conducted in patients with chronic SCI (Guo et al., 2019; Zheng et al., 2017; Huynh et al., 2021; Sun et al., 2017; Ilvesmaki et al., 2017). However, there is limited knowledge regarding the brain’s structural changes, as evaluated by DTI, in an early phase after SCI. This phase is crucial, as adaptive changes of the central nervous system, known as neuroplasticity, are most prominent during the early period following SCI (Henry et al., 2024). For instance, a study in rats with SCI indicates that implementing an exercise routine in the acute phase of the injury maximizes the potential for recovery of function (Brown et al., 2011). Delaying motor training after SCI on the other hand, had a negative impact on the functional recovery in rats (Norrie et al., 2005). It is therefore believed, that early intervention will show the most significant improvements in functional outcomes (Henry et al., 2024; Brown et al., 2011). Understanding the subacute structural brain changes may therefore benefit therapy strategies resulting in better functional outcome.

The present study is – to the best of our knowledge – the first one to investigate structural white matter changes after subacute SCI using both DTI and DKI technique. The aim of our study is to investigate structural changes in the brain using DTI (fractional anisotropy, FA; mean diffusivity, MD) and DKI parameters (kurtosis anisotropy, KA; mean kurtosis, MK) in subacute SCI, and to study whether these changes are associated with clinical outcomes. We hypothesized that DKI captures different properties of the white matter tissue compared to DTI, and correlates with clinical scores such as ISNCSCI and SCIM, and thus is able to link microstructural brain changes in the subacute phase of SCI with clinically meaningful measures of neurological and functional outcomes.

## 2 Materials and methods

### 2.1 Participants and study design

A total of 28 individuals with subacute SCI admitted to the Swiss Paraplegic Centre (Nottwil, Switzerland) for rehabilitation were prospectively recruited between April 1, 2019, and December 31, 2022. Inclusion criteria were as follows: complete or incomplete SCI classified according to the ASIA impairment scale (Asia, Committee ISIS, 2019), lesion level at or below C3 (to exclude ventilator-dependent participants), etiology of the SCI as traumatic or non-traumatic, and age between 18 and 80 years. The MRI measurements and assessments for individuals with subacute SCI were conducted between 70 and 98 days post-injury, as defined by the European Multicenter Study on Spinal Cord Injury (EMSCI) time schedule for acute II (EMSCI, 2018).

The exclusion criteria for individuals with subacute SCI were other known pathologies of the spinal cord/brain (e.g., multiple sclerosis) or progressive neurological disorders, inability to meet the MRI screening requirements (e.g., pacemaker or other electronic devices), severe head trauma as defined by a Glasgow Coma Scale (GCS) of <14, post-traumatic brain injury, and individuals who were ventilator-dependent.

As control group, we included 20 volunteers without SCI, no history of cervical trauma, traumatic brain injury, cervical surgery, no signs of neurological impairment or neurological disease.

### 2.2 Clinical assessment

All individuals with SCI underwent standardized EMSCI assessments, which included the Spinal Cord Independence Measure III (SCIM III) (Catz et al., 1997; Catz et al., 2001; Catz et al., 2001), ASIA impairment scale (AIS) following the International Standards for Neurological Classification of Spinal Cord Injury (ISNCSCI) assessment (Asia, Committee ISIS, 2019; Rupp et al., 2021). The SCIM, ranging from 0 (full impairment) to 100 (no impairment), is a disability scale specifically developed for individuals with SCI, evaluating functional independence in areas such as self-care, respiration, sphincter management, and social integration. The ISNCSCI assessment is a standardized tool used to assess and classify the severity of SCI, including motor and sensory testing, as well as determining the neurological level of injury (Asia, Committee ISIS, 2019; Rupp et al., 2021).

### 2.3 Image acquisition

We acquired the data on a 3 T Philips Achieva scanner (Release 5.4.1, Philips Healthcare, Best, The Netherlands) with a 32-channel head coil. The acquisition protocol consisted of (1) a high-resolution sagittal 3D T1-weighted anatomical sequence [repetition time (TR) = 8 ms, echo time (TE) = 3.7 ms, voxel size = 1 × 1 × 1 mm^3^, acquisition time = 150 s] and (2) a diffusion MRI sequence [TR = 13,000 ms, TE = 80 ms, FOV 240 × 240 × 152 mm^3^, voxel size = 2.5 × 2.5 × 2.5 mm^3^, matrix size 96 × 96, 61 slices, SENSE acceleration factor 2, acquisition time = 900 s] resulting in a total acquisition time of 20 min. The diffusion MRI sequence included 63 diffusion weighted volumes (7 at b = 0 s/mm^2^, 6 at b = 500 s/mm^2^, 20 at b = 1,000 s/mm^2^, and 30 at b = 2000 s/mm^2^).

### 2.4 Diffusion MRI processing

We processed and analyzed the acquired diffusion data with MATLAB R2020a (Mathworks Inc., version 9.8) using ExploreDTI (Leemans et al., 2009) (version 4.8.6) and MRIToolkit^1^ (De Luca, n.d.). The data processing pipeline included signal drift correction (Vos et al., 2017), de-noising with Marchenko-Pastur Principal Component Analysis (MPPCA) (Veraart et al., 2016), Gibbs ringing correction (Perrone et al., 2015), correction for subject motion (Leemans and Jones, 2009), eddy current distortions and EPI deformations. We set the sliding window size used for the MPPCA denoising to 5x5x5 comprising a total of 125 voxels. We estimated the diffusion and kurtosis tensors with linear least square estimator and corrected for unfeasible values with the mean kurtosis (MK)-Curve method (Zhang et al., 2019). In short, the MK-curve method adjusts the measurement at b = 0 s/mm^2^ to ensure that the estimated kurtosis values are plausible (Zhang et al., 2019; Christiaanse et al., 2023). We derived the kurtosis metrics KA and MK and tensor metrics FA and MD as described by Poot et al. (2010) using 1,024 sampling directions. We calculated the DTI metrics from the tensor quantified during the DKI fit, and not with a separate DTI fit.

Outliers outside normal ranges were removed according to the following criteria: valid range for FA and KA was [0:1], for MK [0:3], and for MD [0:0.003] mm/s^2^.

Finally, we extracted regions of interests (ROIs) in the white matter by applying the Statistical Parametric Mapping (SPM) based Cat12 toolbox (Gaser and Dahnke, 2016) to the 3D T1w in order to conduct brain segmentation using the ICBM DTI-81 white matter atlas (Rezende et al., 2019).

### 2.5 Statistical analysis

Statistical analyses were performed in R (version 4.1.3). FA, KA, MD and MK measures in 17 predefined ROIs (Supplementary Table S1) were compared individually between subjects with SCI and without SCI using unpaired t-tests. Given the large number of included regions, we focused on a subset of 17 ROIs, which were selected based on prior studies reporting significant DTI changes following SCI (Guo et al., 2019; Zheng et al., 2017; Huynh et al., 2021; Sun et al., 2017; Ilvesmaki et al., 2017). Benjamini and Hochberg (BH) correction was applied to correct for multiple comparisons across the 17 regions for each diffusion metric separately (Benjamini and Hochberg, 2018). The threshold for statistical significance was set at p_BH_ = 0.05. Effect sizes were calculated using the absolute value of Cohen’s d. Spearman correlations between clinical variables including total SCIM, total ASIA pinprick, light touch and motor scores were conducted with the DTI/DKI measures of regions with significant results in the group comparison. *p*-values were corrected for multiple comparisons using Benjamini and Hochberg correction. Subgroup analyses in individuals with SCI were conducted between different lesion levels (cervival, thoracic, lumbar) as well as ASIA impairment scale (AIS) levels across the DTI and DKI measures in the 17 regions using a one-way ANOVA (Supplementary material).

## 3 Results

A total of 28 individuals (20 men and 8 women, with a mean age of 47.9 ± 15.9 years) with subacute SCI completed the study protocol and underwent an MRI. The clinical characteristics of the SCI group are displayed in Table 1. Twenty healthy controls (9 men, 11 women, mean age 41 ± 12.61 years) underwent one MRI. The two groups did not significantly differ with respect to age (*p* = 0.202, W = 218) and sex (*p* = 0.065, $\chi^{2}$=3.41). The distribution of the DTI and DKI measures across the regions of interest in individuals with subacute SCI and non-injured controls is shown in the Supplementary Figures S1–S4.

**Table 1:** Detailed demographics of participants with subacute SCI at timepoint 1.

SCI subject	Age (years)	Sex	Cause of injury	Level of motor/sensory impairment neurological lesion level	ASIA	Severity	Time between SCI and MRI (days)
1	68	F	Surgery	Th3	D	Incomplete, Paraplegic	89
2	75	F	Spinalis anterior syndrome	C4	C	Incomplete, Tetraplegic	81
3	23	F	Ischemia	C4	D	Incomplete, Tetraplegic	98
4	67	M	Trauma	Th6	A	Complete, Paraplegic	92
5	74	M	Trauma	Th5	A	Incomplete, Paraplegic	82
6	51	M	Barotrauma	Th5	D	Incomplete, Paraplegic	93
7	68	M	Trauma	C6	D	Incomplete, Tetraplegic	71
8	41	M	Trauma	Th12	D	Incomplete, Paraplegic	73
9	42	M	Trauma	C5	A	Complete, Tetraplegic	73
10	55	M	Trauma	C5	A	Complete, Tetraplegic	88
11	55	M	Trauma	C5	D	Incomplete, Tetraplegic	78
12	61	M	Trauma	Th9	C	Incomplete, Paraplegic	73
13	23	M	Trauma	L1	D	Incomplete, Paraplegic	86
14	47	F	Trauma	L2	C	Incomplete, Paraplegic	75
15	53	M	Trauma	C4	D	Incomplete, Tetraplegic	91
16	38	M	Trauma	L1	A	Complete, Paraplegic	93
17	38	M	Trauma	Th4	A	Complete, Paraplegic	85
18	25	M	Trauma	C4	A	Complete, Tetraplegic	98
19	46	M	Trauma	Th3	A	Complete, Paraplegic	71
20	55	M	Trauma	L2	C	Incomplete, Paraplegic	87
21	31	F	Trauma	L1	B	Incomplete, Paraplegic	94
22	35	M	Trauma	L1	C	Incomplete, Paraplegic	95
23	37	M	Trauma	Th4	A	Complete, Paraplegic	88
24	40	F	Trauma	L1	A	Complete, Paraplegic	89
25	23	F	Trauma	Th12	A	Complete, Paraplegic	91
26	42	F	Trauma	Th7	A	Complete, Paraplegic	74
27	67	M	Trauma	C6	D	Incomplete, Tetraplegic	89
28	22	M	Trauma	Th4	A	Complete, Paraplegic	94

**Figure 1:**
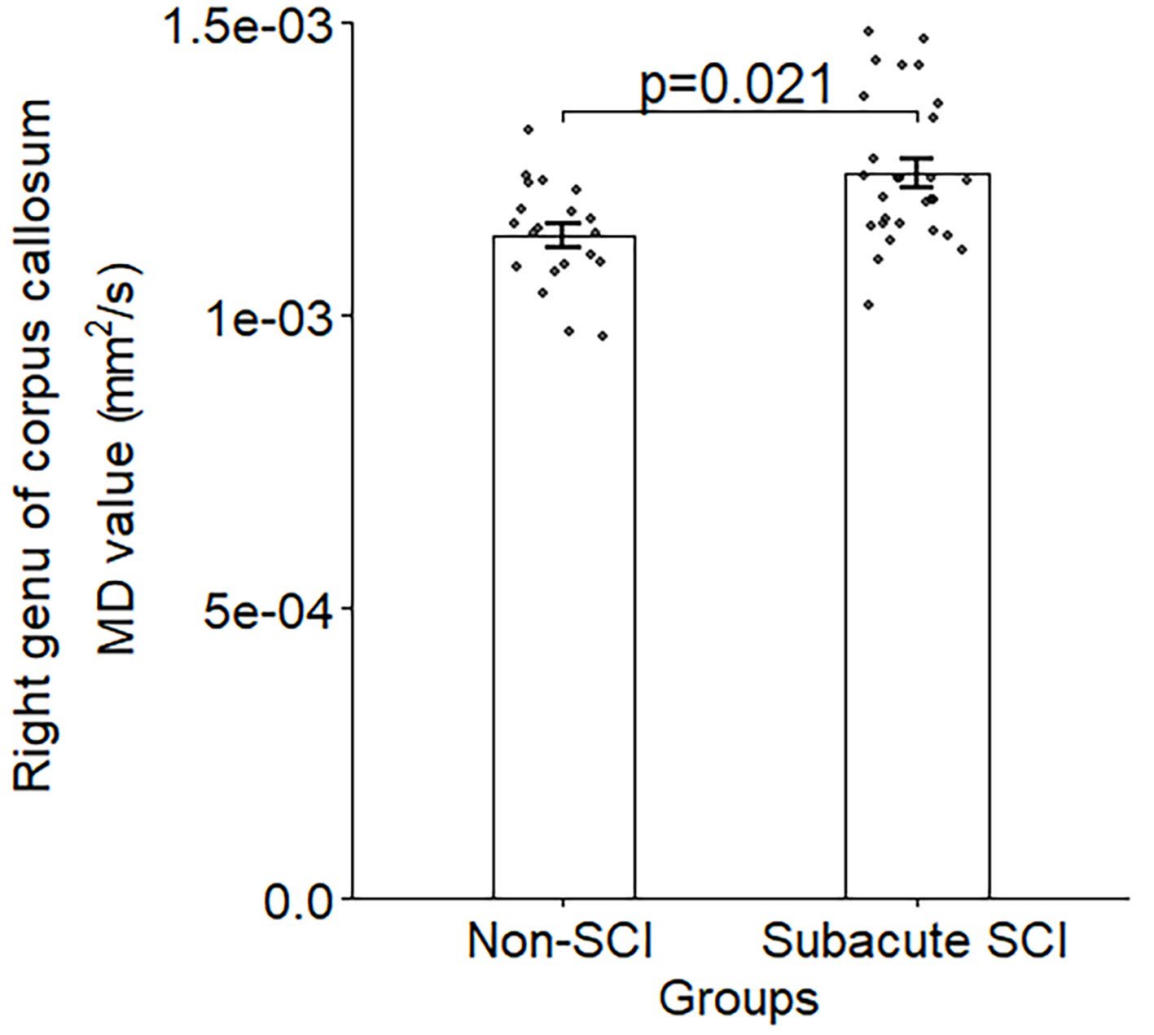
Bar plot illustrating the significant difference in mean diffusivity (MD) between subacute SCI and non-injured controls in the right genu of the corpus callosum. Single data points illustrate the MD values in individual subjects. The error bars indicate the standard error of the mean.

**Figure 2:**
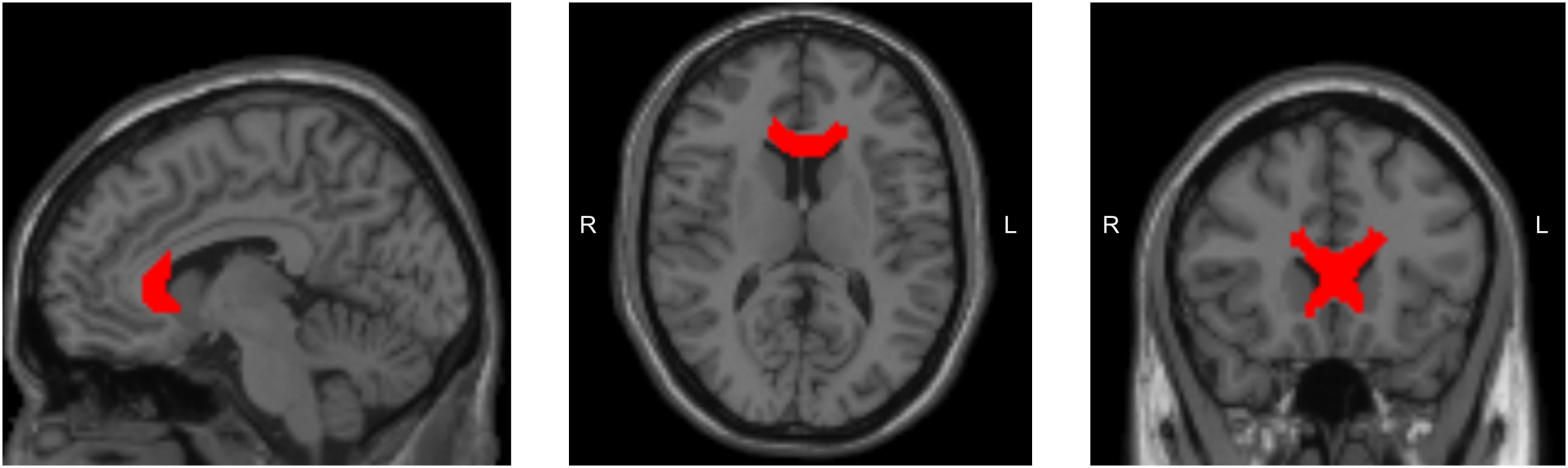
Brain image illustrating the location of the ROI for the genu of the corpus callosum.

**Figure 3:**
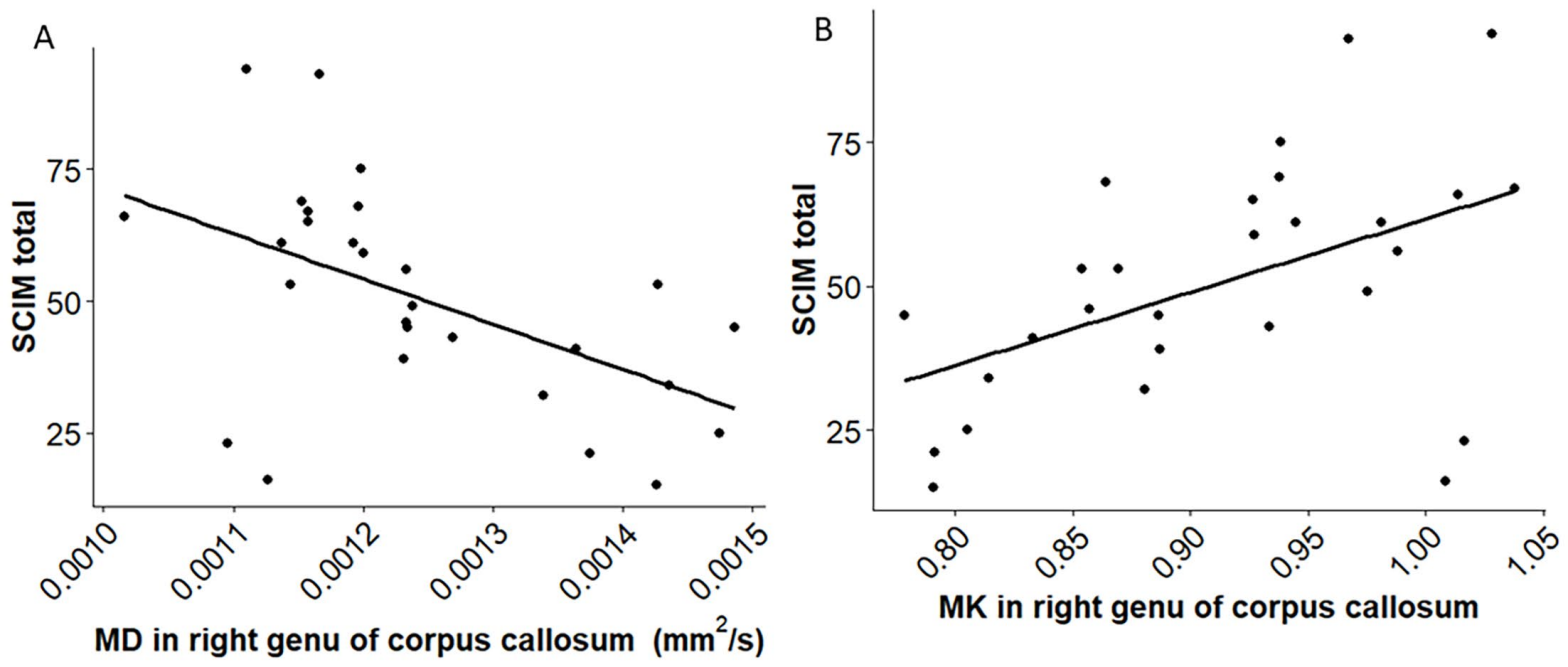
Scatterplot illustrating the association between MD and MK with the spinal cord independence measure (SCIM). **(A)** The negative correlation (*r* = −0.51, p_BH_ = 0.022) between the SCIM and MD in the right genu of the corpus callosum (rCCGen) in individuals with spinal cord injury. **(B)** The positive correlation between SCIM and MK in rCCGen (*r* = 0.482, p_BH_ = 0.038).

After multiple comparison correction, the right genu of the corpus callosum showed significant differences in MD (p_BH_ = 0.021, cohen’s d = 0.955) between SCI and healthy controls (Figures 1, 2; Supplementary Table S2) with higher MD in SCI (Supplementary Figure S3). In contrast, MK in the same region showed a trend to lower values in SCI compared to healthy controls with a significant difference before correction (Supplementary Figure S4; Supplementary Table S2). Other regions also exhibited significant differences before correction, none remained significant after correction for multiple comparisons (Supplementary Tables S2, S3). No significant differences in FA and KA were observed across different lesion levels and AIS grades (Supplementary Tables S4–S7). A significant negative correlation was observed between MD in the right genu of the corpus callosum and the SCIM total score (*r* = −0.51, p_BH_ = 0.022) in individuals with SCI (Figure 3A; Supplementary Table S4). In contrast, a significant positive correlation was found between MK and the SCIM total score, with higher MK values in the right genu of the corpus callosum being associated with higher SCIM total scores (*r* = 0.482, pBH = 0.038) (Figure 3B; Supplementary Table S5). The correlations between MD and MK in the right genu of the corpus callosum with the ASIA pinprick, light touch and motor scores were not significant (Supplementary Tables S8, S9).

## 4 Discussion

We present a study analyzing structural brain changes after SCI with a comparison between DTI and DKI measurements of the brain and correlation with clinical variables in individuals with subacute SCI. We observed a significant difference in MD in the right genu of the corpus callosum between individuals with subacute SCI and non-injured controls, with SCI individuals showing higher MD compared to healthy controls. Additionally, in the right genu of the corpus callosum, a negative correlation was found between MD and the SCIM total score, as well as a positive correlation between MK and the total SCIM score. Our findings are consistent with previous studies that reported increased MD in the corpus callosum in individuals with SCI (Guo et al., 2019; Ilvesmaki et al., 2017).

MD is a scalar metric that reflects the average diffusivity of water molecules within a voxel (Le Bihan et al., 1986). At the microstructural level, MD is influenced by several biological factors, including cell membrane integrity, intracellular and extracellular volume fractions, and the density and organization of cellular and subcellular components (Le Bihan et al., 2001), but also perfusion (De Luca et al., 2018) and partial volume effects. In healthy white matter, the presence of intact axonal membranes and myelin sheaths restricts the free diffusion of water molecules, hindering MD to much lower values than those of freely diffusing water. When tissue structural integrity is impaired by processes such as axon degeneration, myelin breakdown, edema, or cell death, the barriers that typically hinder and restrict diffusion are reduced (Song et al., 2003). This results in an increase in the extracellular space and a corresponding rise in water mobility, which is reflected by an increase in MD (Stebbins, 2010; Schmierer et al., 2007). Mean kurtosis (MK), on the other hand, is derived from DKI, an extension of DTI that quantifies the non-Gaussianity of water diffusion (Jensen et al., 2005; Jensen and Helpern, 2010). The assumption of Gaussian diffusion relies on a uniform medium, yet biological tissues are structurally heterogeneous, containing numerous microstructural barriers, such as cell membranes and organelles, that influence water motion in anisotropic and non-linear ways (Tuch et al., 2003). MK quantifies the degree to which this diffusion deviates from a Gaussian profile, making it a sensitive marker for microstructural complexity and tissue heterogeneity. High MK values are typically observed in healthy, well-organized white matter due to the abundance of microstructural obstacles that restrict diffusion. A decrease in MK therefore suggests a loss of tissue complexity, which may result from the breakdown of structural barriers due to demyelination, reduction in axonal diameter, or loss of cellular organization due to gliotic scarring or neuroinflammation.

In the context of SCI, secondary changes extend beyond the primary lesion site and can impact distant supraspinal structures through Wallerian degeneration and systemic inflammatory responses (Ziegler et al., 2018; Bloom et al., 2020; Anjum et al., 2020). These processes can lead to white matter atrophy and demyelination, including major commissural pathways such as the corpus callosum (Freund et al., 2013; Ziegler et al., 2018; Buss and Schwab, 2003). Elevated MD in this region likely reflects the cumulative effects of axonal loss and myelin degradation, consistent with prior histopathological and neuroimaging findings in SCI and other neurodegenerative conditions (Le Bihan et al., 1986; Le Bihan et al., 2001; Schmierer et al., 2007; Garg et al., 2015).

In our study, the simultaneous increase in MD and a trend toward decreased MK in the right genu of the corpus callosum among individuals with SCI is consistent with microstructural changes after SCI. From a neurobiological perspective, the observed diffusion changes can presumably be attributed to a combination of anterograde degeneration of corticospinal tracts, cortical reorganization, and inflammation-mediated demyelination, all of which contribute to alterations in white matter microstructure. The pattern of increased MD and reduced MK may therefore serve as a sensitive imaging marker for tracking disease progression, assessing treatment effects, or predicting functional outcomes in individuals with SCI.

In previous literature (Cunningham et al., 2019; Friedrich et al., 2020; Dong et al., 2023), differences in DTI measures have been observed after SCI in similar brain regions as in our study. For instance, the genu of the corpus callosum has been associated with functional motor ability of the upper limb, with higher FA indicating better ability (Cunningham et al., 2019). Beyond motor function, however, changes in the corpus callosum have also been linked to sensory and pain-related processes. For example, a previous study observed changes in the corpus callosum in individuals with subacute SCI and neuropathic pain, which may be associated with disrupted somatosensory information processing or interhemispheric communication. These alterations could trigger excitatory activity, potentially leading to cortical hyperexcitability linked to the perception of neuropathic pain (Dong et al., 2023). Alterations in parallel and perpendicular diffusivities in the corpus callosum and corticospinal tract were also associated with interhemispheric reorganization in motor regions and motor impairments in stroke patients (Wang et al., 2012). The corpus callosum has been widely recognized as a critical structure for interhemispheric integration, facilitating the exchange of motor, sensory, and cognitive information between the brain’s hemispheres (Friedrich et al., 2020). This integrative function is particularly important for bimanual coordination, where the timing and execution of movements by both hands must be precisely aligned. Evidence from both clinical and neuroimaging studies across various neurological and developmental conditions highlights the critical role of the corpus callosum in planning and synchronizing bimanual actions (Pauwels and Gooijers, 2023). Structural alterations in the genu of the right corpus callosum after SCI, reflected by increased MD, were linked to poorer functional outcomes, as evidenced by a negative correlation between MD and SCIM scores. In contrast, the positive correlation between SCIM and mean kurtosis MK indicates that higher microstructural complexity is associated with better recovery, underscoring the critical role of the corpus callosum in interhemispheric communication and the coordination of sensory and motor functions essential for functional independence, as captured by the SCIM.

The changes in the corpus callosum in our study may therefore reflect structural and functional plasticity in the nervous system in response to the injury (Ding et al., 2005; Gatto, 2020).

The total SCIM score helps predict how likely and how difficult it will be for a person to become independent in specific daily activities. Hence, the current total SCIM score can be used as a guide for assessing progress in daily living skills. Additionally, higher total scores—and the chances of success with each individual activity—can help healthcare providers plan more effective rehabilitation strategies, based on how challenging each task is for the person (Unai et al., 2019). Our findings may have a clinical implication for prognostic value, for instance, they may support the identification of imaging-based biomarkers of SCI, to be monitored throughout the subacute phase. With regards to prognosis, brain imaging has already shown functional and structural changes following SCI (Freund et al., 2019), and it has been highlighted that, while clinical recovery stops improving after 2 years post-injury, both macroscopic and microstructural changes in the brain continue, with the highest predictive validity in relation to clinical outcome at the level of the spinal cord, brainstem, and cortex over the first 6 months (Ziegler et al., 2018; Seif et al., 2018). Our results suggests that early structural brain changes after SCI, as depicted by the elevated MD in SCI and by the correlation between MD and MK with the SCIM score, take place already during the subacute phase. With regards to therapy, treatment of SCI-induced comorbidities remains difficult due to the incomplete understanding of its underlying mechanisms (Shiao and Lee-Kubli, 2018). Detecting changes in the brain after SCI requires the use of more advanced and sensitive techniques such as DKI. Our study is, to the best of our knowledge, among the first ones to apply DKI to the brain of individuals with SCI, and therefore only limited literature is available in the field. For instance, in a study comparing patients with early-clinical-stage cervical spondylotic myelopathy (CSM) to healthy controls, decreased MK values were found in white matter of the cervical spinal cord in the CSM group (Liu et al., 2020). Another study showed significant differences in MK and radial kurtosis in the spinal cord of pediatric individuals with chronic SCI compared to healthy controls (Conklin et al., 2016). The latter study suggested that DKI is a promising technique to characterize microstructural changes in SCI at the level of the spinal cord. One novel aspect of our study is that, while previous research has focused on chronic SCI, we included subjects in the subacute phase, and demonstrated that there is a positive correlation between MK and SCIM between 70 and 98 days after injury.

In our study, significant changes between the two groups were detected for MD with a trend toward decreased MK. This may be because these techniques measure different properties. While DKI measures are generally more sensitive to structural changes, these changes may take longer to develop to reach a certain threshold, making them detectable by MK only after a certain period. Previous studies in SCI (Guo et al., 2019; Zheng et al., 2017; Huynh et al., 2021) mostly included individuals with chronic injuries which may have made it easier to detect broader changes in the brain. Other studies have demonstrated that DKI is highly sensitive in detecting pathology in both gray matter (Zhuo et al., 2012) and white matter (Guglielmetti et al., 2016), and is able to capture both anisotropic and isotropic diffusion, suggesting that DKI has the potential to serve as an early-stage biomarker for various neurodegenerative disorders (Arab et al., 2018). Moreover, DKI allows for the assessment of isotropic structures, including the cortex and basal ganglia, which is an important limitation of DTI (Steven et al., 2014). It also enables the detection of crossing fibers, which results in a substantial improvement relative to DTI, as up to 90% of white matter contains two or more fiber populations crossing at different levels (Jeurissen et al., 2013), and this information is crucial when investigating tractography throughout regions with complex fiber bundle geometries (Glenn et al., 2016).

As this is the first study to investigate brain alterations with DKI measures in subacute SCI, further studies need to be conducted to investigate potential correlations of white matter microstructural properties with other clinical variables to understand their relationship with symptoms present in SCI. DKI shows potential for monitoring structural brain changes, assessing therapeutic response, and detecting early signs of clinical deterioration. Additionally, it may support the development of personalized treatment strategies by identifying region-specific changes, particularly in motor and sensory areas, that reflect adaptive plasticity. These insights could help clinicians determine the most effective timing and intensity of interventions to optimize functional outcomes.

Although DKI provides additional sensitivity to microstructural changes, it also presents certain limitations compared to DTI. DKI generally employs higher b-values (up to 3,000 s/mm^2^) than DTI, requiring stronger diffusion gradients. Because clinical MRI systems are limited in their maximum gradient amplitude, the desired diffusion weighting is often achieved by prolonging the gradient duration. This can lead to an increased echo time, which may reduce the signal-to-noise ratio, extend the total scan time, and consequently increase sensitivity to motion artifacts and the presence of outliers (Li et al., 2014). In clinical settings, image quality may also be compromised by spasticity, which is common after SCI and may be more likely to affect scans with longer acquisition times. The higher model complexity of DKI inherently leads to higher sensitivity to signal noise (Glenn et al., 2015). Additionally, DKI involves greater post-processing complexity due to the nonlinear fitting of higher-order diffusion models, which demands extensive computational resources and rigorous preprocessing steps such as denoising and artifact correction. This complexity further elevates the risk of fitting instabilities and model overfitting, necessitating careful optimization to achieve robust and reliable parameter estimation. DKI metrics can also be more difficult to interpret in heterogeneous tissues because the diffusion signal reflects a complex combination of microstructural environments within each voxel. This complexity leads to overlapping contributions from different tissue types and structures, making it challenging to attribute changes in kurtosis measures to specific biological processes. Another limitation of the study is the limited number of SCI subjects and heterogeneous SCI sample in terms of lesion and AIS level. We conducted subgroup analyses to investigate potential differences in DTI and DKI measures, but we did not find any significant results, which could also be attributed to the small sample size within each subgroup. Further research should examine the effect of lesion and AIS level on diffusion metrics in individuals with SCI. Moreover, there was a trend toward significance in the sex ratio between the SCI group and the controls. However, due to the small sample size, the study may be underpowered for including sex as an additional covariate in the model, and therefore future studies should include more participants and better matched groups.

## 5 Conclusion

Our study demonstrated that DTI and DKI can assess structural brain changes during the subacute phase following SCI and show meaningful correlations with clinical outcome measures. The genu of the corpus callosum showed significant changes in MD between individuals with SCI and non-injured controls, which may be related to phenomena of maladaptive plasticity following the injury. Structural tissue properties, indicated by MD and MK, were correlated with functional independence, as reflected by the SCIM scores. With the appropriate transfer from research to clinical applications, DKI may be used in clinical practize to monitor plastic changes in the brain after SCI, as well as to develop future treatment techniques.

## Data Availability

The datasets presented in this article are not readily available because the data that support the findings of this study are available from the corresponding author upon reasonable request. Requests to access the datasets should be directed to ernst.christiaanse@paraplegie.ch.

## Footnotes

1
www.mritoolkit.org
