# Supplementary material for: Structural brain changes in subacute spinal cord injury: an analysis of diffusion kurtosis imaging and diffusion tensor imaging metrics with clinical correlation
Source: Front Neurosci. 2025 Nov 28;19:1652416. doi: 10.3389/fnins.2025.1652416 (PMC12698660; doi:10.3389/fnins.2025.1652416)
Supplement: Supplementary file 1 [file Data_Sheet_1.docx]

**Supplementary material**

**Structural brain changes in subacute spinal cord injury: an analysis of diffusion kurtosis imaging and diffusion tensor imaging metrics with clinical correlation**

Table S1: Selected ROIs based on previous literature. All regions are extracted from the ICBM-DTI-81 atlas.

| ROI | Reference |
| --- | --- |
| Left genu corpus callosum | Guo et al (1)  Huynh et al (2)  Ilvesmäki et al (3) |
| Right genu corpus callosum | Guo et al (1)  Huynh et al (2)  Ilvesmäki et al (3) |
| Left body corpus callosum | Guo et al (1)  Huynh et al (2)  Ilvesmäki et al (3) |
| Right body corpus callosum | Guo et al (1)  Huynh et al (2)  Ilvesmäki et al (3) |
| Left anterior corona radiata | Guo et al (1)  Ilvesmäki et al (3) |
| Right anterior corona radiata | Guo et al (1)  Ilvesmäki et al (3) |
| Left posterior corona radiata | Guo et al (1)  Ilvesmäki et al (3) |
| Right posterior corona radiata | Guo et al (1)  Ilvesmäki et al (3) |
| Left superior corona radiata | Guo et al (1)  Ilvesmäki et al (3) |
| Right superior corona radiata | Guo et al (1)  Ilvesmäki et al (3) |
| Left posterior thalamic radiation | Guo et al (1)  Ilvesmäki et al (3)  Huynh et al (2) |
| Right posterior thalamic radiation | Guo et al (1)  Ilvesmäki et al (3)  Huynh et al (2) |
| Left Cingulate and Cingulum Gyrus | Guo et al (1) |
| Right Cingulate and Cingulum Gyrus | Guo et al (1) |
| Right superior cerebellar peduncle | Ilvesmäki et al (3)  Sun et al (4) |
| Right Middle Cerebellar Peduncle | Ilvesmäki et al (3)  Sun et al (4) |
| Left super longitudinal fascicle | Zheng et al (5) |


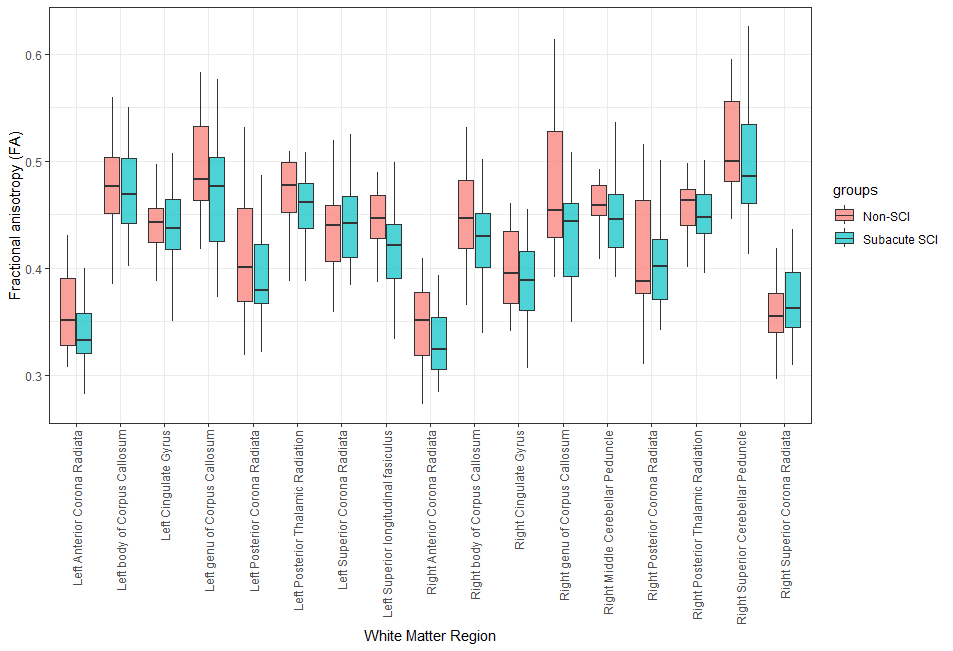


Figure S1: Boxplots showing distribution of fractional anisotropy (FA) values across the 17 regions in individuals with subacute spinal cord injury and non-injured controls.


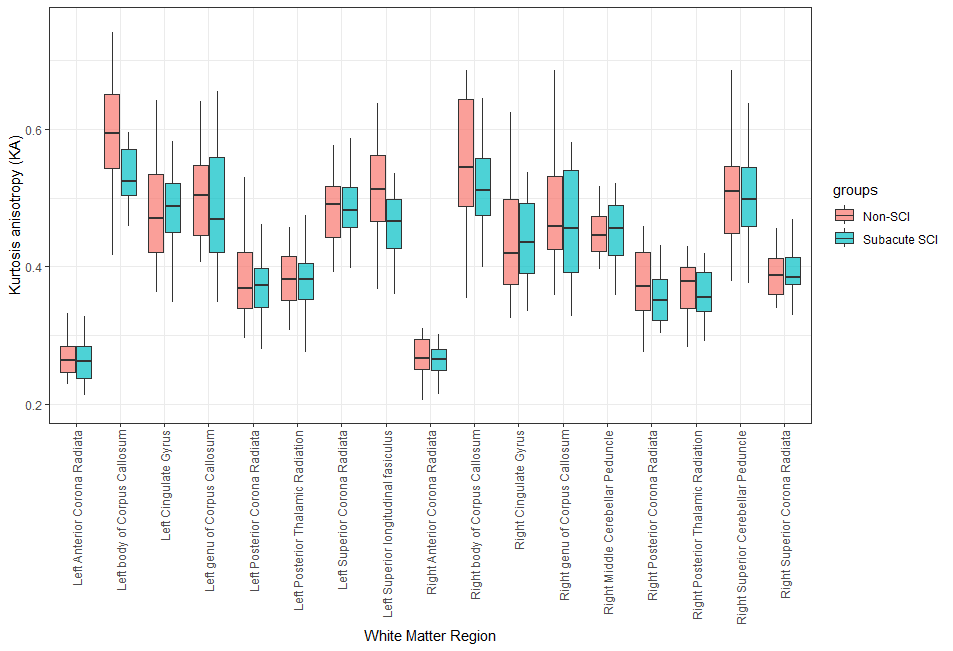
Figure S2: Boxplots showing distribution of kurtosis anisotropy (KA) values across the 17 regions in individuals with subacute spinal cord injury and non-injured controls.


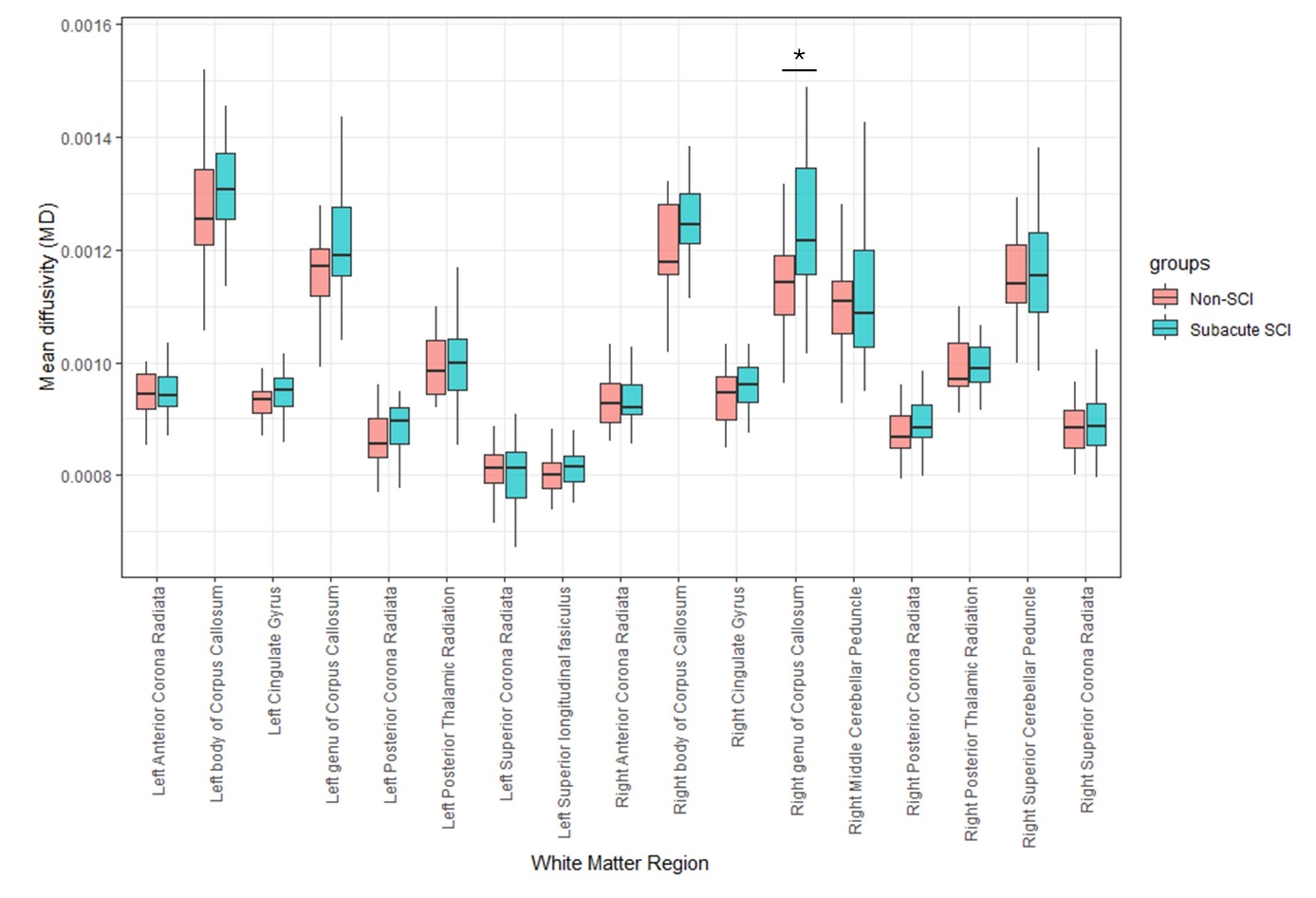
Figure S3: Boxplots showing distribution of mean diffusivity (MD) values across the 17 regions in individuals with subacute spinal cord injury and non-injured controls. An asterisk indicates a significant difference between the two groups after correcting for multiple comparisons using Benjamini & Hochberg correction.


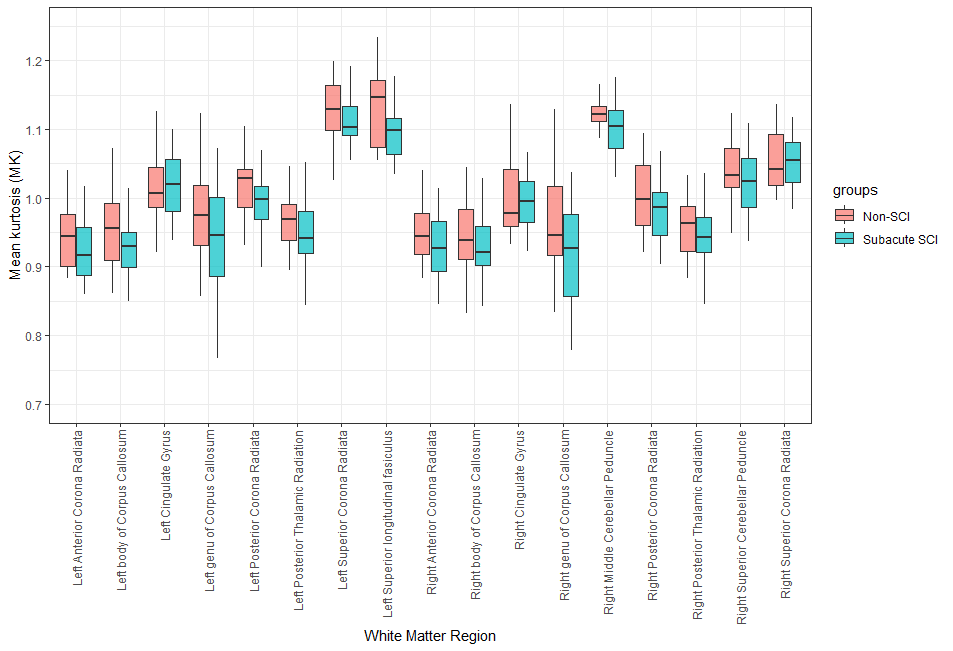
Figure S4: Boxplots showing distribution of mean kurtosis (MK) values across the 17 regions in individuals with subacute spinal cord injury and non-injured controls.

Table S2 : Overview of the uncorrected p-value, corrected p-value, and Cohen's d for MK and MD metric across each region of interest for the group comparison subacute spinal cord injury vs. non-injured controls.

| ROI | MD uncorrected p-value | MD adjusted p-value | MD Cohen’s_d | MK uncorrected p-value | MK adjusted p-value | MK Cohen’s d |
| --- | --- | --- | --- | --- | --- | --- |
| Left genu of corpus callosum | 0,031 | 0,173 | 0,627 | 0,123 | 0,284 | 0,449 |
| Right genu of corpus callosum | 0,001 | **0,021** | 0,955 | 0,02 | 0,253 | 0,699 |
| Left body of corpus callosum | 0,37 | 0,575 | 0,285 | 0,125 | 0,284 | 0,427 |
| Right body of corpus callosum | 0,016 | 0,14 | 0,722 | 0,134 | 0,284 | 0,46 |
| Left anterior corona radiata | 0,372 | 0,575 | 0,249 | 0,186 | 0,292 | 0,392 |
| Right anterior corona radiata | 0,766 | 0,766 | 0,086 | 0,189 | 0,292 | 0,394 |
| Left posterior corona radiata | 0,088 | 0,373 | 0,533 | 0,055 | 0,284 | 0,596 |
| Right posterior corona radiata | 0,246 | 0,524 | 0,347 | 0,301 | 0,394 | 0,312 |
| Left superior corona radiata | 0,713 | 0,766 | 0,104 | 0,344 | 0,418 | 0,293 |
| Right superior corona radiata | 0,552 | 0,671 | 0,171 | 0,713 | 0,757 | 0,111 |
| Left posterior thalamic radiation | 0,743 | 0,766 | 0,092 | 0,079 | 0,284 | 0,513 |
| Right posterior thalamic radiation | 0,333 | 0,575 | 0,271 | 0,174 | 0,292 | 0,404 |
| Left Cingulate and Cingulum Gyrus | 0,232 | 0,524 | 0,367 | 0,825 | 0,825 | 0,068 |
| Right Cingulate and Cingulum Gyrus | 0,135 | 0,458 | 0,453 | 0,428 | 0,485 | 0,258 |
| Right superior cerebellar peduncle | 0,491 | 0,642 | 0,197 | 0,279 | 0,394 | 0,316 |
| Right Middle Cerebellar Peduncle | 0,457 | 0,642 | 0,216 | 0,111 | 0,284 | 0,453 |
| Left super longitudinal fascicle | 0,219 | 0,524 | 0,383 | 0,03 | 0,253 | 0,706 |

Table S3: Overview of the uncorrected p-value, corrected p-value, and Cohen's d for FA and KA across each region of interest for the group comparison subacute spinal cord injury vs. non-injured controls.

| ROI | FA Uncorrected p-value | FA adjusted p-value | FA Cohen’s d | KA Uncorrected p-value | KA adjusted p-value | KA Cohen’s d |
| --- | --- | --- | --- | --- | --- | --- |
| Left genu of corpus callosum | 0.094 | 0.255 | 0.5 | 0.586 | 0.896 | 0.155 |
| Right genu of corpus callosum | 0.011 | 0.185 | 0.835 | 0.325 | 0.896 | 0.297 |
| Left body of corpus callosum | 0.814 | 0.844 | 0.07 | 0.148 | 0.896 | 0.467 |
| Right body of corpus callosum | 0.12 | 0.255 | 0.482 | 0.247 | 0.896 | 0.357 |
| Left anterior corona radiata | 0.035 | 0.255 | 0.67 | 0.865 | 0.896 | 0.05 |
| Right anterior corona radiata | 0.072 | 0.255 | 0.569 | 0.618 | 0.896 | 0.15 |
| Left posterior corona radiata | 0.11 | 0.255 | 0.502 | 0.427 | 0.896 | 0.253 |
| Right posterior corona radiata | 0.588 | 0.666 | 0.17 | 0.445 | 0.896 | 0.23 |
| Left superior corona radiata | 0.376 | 0.481 | 0.255 | 0.896 | 0.896 | 0.039 |
| Right superior corona radiata | 0.213 | 0.363 | 0.366 | 0.666 | 0.896 | 0.126 |
| Left posterior thalamic radiation | 0.108 | 0.255 | 0.478 | 0.559 | 0.896 | 0.168 |
| Right posterior thalamic radiation | 0.396 | 0.481 | 0.26 | 0.783 | 0.896 | 0.078 |
| Left Cingulate and Cingulum Gyrus | 0.844 | 0.844 | 0.057 | 0.892 | 0.896 | 0.043 |
| Right Cingulate and Cingulum Gyrus | 0.27 | 0.396 | 0.33 | 0.589 | 0.896 | 0.16 |
| Right superior cerebellar peduncle | 0.28 | 0.396 | 0.311 | 0.763 | 0.896 | 0.089 |
| Right Middle Cerebellar Peduncle | 0.17 | 0.321 | 0.403 | 0.818 | 0.896 | 0.066 |
| Left super longitudinal fascicle | 0.097 | 0.255 | 0.508 | 0.052 | 0.886 | 0.643 |

**Subgroup analysis across lesion levels**

Among the individuals with subacute SCI there were 9 participants with cervical, 7 with lumbar and 12 with thoracic SCI. In order to investigate potential differences across lesion levels in the DTI and DKI measures, a one-way ANOVA was applied. Benjamini & Hochberg (BH) correction was applied to correct for multiple comparisons across the 17 regions for each diffusion metric separately (6). The threshold for statistical significance was set at p_BH_=0.05. The detailed results, including F-values, are displayed in Tables S4 and S5.

There was no significant difference in any of the measures and regions between the different lesion levels. Due to the small sample size within each subgroup, it cannot be ruled out that the analysis may be underpowered, which is an important consideration when interpreting the results.

| Table S4: Overview of the uncorrected p-value, corrected p-value, and F-values for FA and KA across each region of interest for the group comparison across lesion levels (cervical, thoracic, lumbar) in individuals with subacute spinal cord injury.ROI | FA Uncorrected p-value | FA adjusted p-value | FA F-value | KA Uncorrected p-value | KA adjusted p-value | KA F-value |
| --- | --- | --- | --- | --- | --- | --- |
| Left genu of corpus callosum | 0.599 | 0.966 | 0.523 | 0.806 | 0.895 | 0.217 |
| Right genu of corpus callosum | 0.778 | 0.966 | 0.254 | 0.786 | 0.895 | 0.244 |
| Left body of corpus callosum | 0.702 | 0.966 | 0.359 | 0.132 | 0.895 | 2.245 |
| Right body of corpus callosum | 0.966 | 0.966 | 0.034 | 0.569 | 0.895 | 0.578 |
| Left anterior corona radiata | 0.935 | 0.966 | 0.067 | 0.589 | 0.895 | 0.541 |
| Right anterior corona radiata | 0.951 | 0.966 | 0.05 | 0.656 | 0.895 | 0.428 |
| Left posterior corona radiata | 0.965 | 0.966 | 0.036 | 0.397 | 0.895 | 0.958 |
| Right posterior corona radiata | 0.574 | 0.966 | 0.568 | 0.369 | 0.895 | 1.039 |
| Left superior corona radiata | 0.205 | 0.966 | 1.692 | 0.614 | 0.895 | 0.498 |
| Right superior corona radiata | 0.054 | 0.911 | 3.298 | 0.727 | 0.895 | 0.323 |
| Left posterior thalamic radiation | 0.464 | 0.966 | 0.793 | 0.492 | 0.895 | 0.73 |
| Right posterior thalamic radiation | 0.73 | 0.966 | 0.318 | 0.269 | 0.895 | 1.386 |
| Left Cingulate and Cingulum Gyrus | 0.89 | 0.966 | 0.117 | 0.339 | 0.895 | 1.131 |
| Right Cingulate and Cingulum Gyrus | 0.737 | 0.966 | 0.309 | 0.859 | 0.895 | 0.153 |
| Right superior cerebellar peduncle | 0.293 | 0.966 | 1.29 | 0.895 | 0.895 | 0.112 |
| Right Middle Cerebellar Peduncle | 0.408 | 0.966 | 0.93 | 0.646 | 0.895 | 0.445 |
| Left super longitudinal fascicle | 0.469 | 0.966 | 0.781 | 0.579 | 0.895 | 0.56 |

Table S5: Overview of the uncorrected p-value, corrected p-value, and F-values for MD and MK across each region of interest for the group comparison across lesion levels (cervical, thoracic, lumbar) in individuals with subacute spinal cord injury.

| ROI | MD Uncorrected p-value | MD adjusted p-value | MD F-value | MK Uncorrected p-value | MK adjusted p-value | MK F-value |
| --- | --- | --- | --- | --- | --- | --- |
| Left genu of corpus callosum | 0.177 | 0.734 | 1.855 | 0.556 | 0.819 | 0.601 |
| Right genu of corpus callosum | 0.209 | 0.734 | 1.669 | 0.541 | 0.819 | 0.629 |
| Left body of corpus callosum | 0.942 | 0.997 | 0.06 | 0.251 | 0.819 | 1.468 |
| Right body of corpus callosum | 0.297 | 0.734 | 1.274 | 0.264 | 0.819 | 1.408 |
| Left anterior corona radiata | 0.363 | 0.734 | 1.054 | 0.581 | 0.819 | 0.554 |
| Right anterior corona radiata | 0.376 | 0.734 | 1.018 | 0.723 | 0.819 | 0.329 |
| Left posterior corona radiata | 0.997 | 0.997 | 0.003 | 0.126 | 0.819 | 2.252 |
| Right posterior corona radiata | 0.613 | 0.997 | 0.499 | 0.104 | 0.819 | 2.478 |
| Left superior corona radiata | 0.691 | 0.997 | 0.375 | 0.563 | 0.819 | 0.587 |
| Right superior corona radiata | 0.389 | 0.734 | 0.981 | 0.311 | 0.819 | 1.223 |
| Left posterior thalamic radiation | 0.841 | 0.997 | 0.174 | 0.699 | 0.819 | 0.364 |
| Right posterior thalamic radiation | 0.963 | 0.997 | 0.038 | 0.52 | 0.819 | 0.672 |
| Left Cingulate and Cingulum Gyrus | 0.246 | 0.734 | 1.485 | 0.675 | 0.819 | 0.399 |
| Right Cingulate and Cingulum Gyrus | 0.223 | 0.734 | 1.592 | 0.803 | 0.853 | 0.221 |
| Right superior cerebellar peduncle | 0.192 | 0.734 | 1.765 | 0.495 | 0.819 | 0.723 |
| Right Middle Cerebellar Peduncle | 0.909 | 0.997 | 0.096 | 0.936 | 0.936 | 0.066 |
| Left super longitudinal fascicle | 0.794 | 0.997 | 0.233 | 0.353 | 0.819 | 1.089 |

**Subgroup analysis across ASIA levels**

Among the individuals with subacute SCI there were 13 participants with American spinal injury association (ASIA) Impairment Scale (AIS) level A, 1 with AIS level B, 5 with AIS level C and 9 with AIS level D. In order to investigate potential differences across AIS levels in the DTI and DKI measures, a one-way anova was applied. As there was only one individuals with AIS level B, this person was discarded from this analysis. Benjamini & Hochberg (BH) correction was applied to correct for multiple comparisons across the 17 regions for each diffusion metric separately (6). The threshold for statistical significance was set at p_BH_=0.05. The detailed results including F-values are displayed in Tables S6 and S7.

There was no significant difference in any of the measures and regions between the different AIS levels. Due to the small sample size within each subgroup, it cannot be ruled out that the analysis may be underpowered, which should be considered when interpreting the results.

Table S6: Overview of the uncorrected p-value, corrected p-value, and F-values for FA and KA across each region of interest for the group comparison across American spinal injury association (ASIA) Impairment Scale (AIS) levels in individuals with subacute spinal cord injury.

| ROI | FA Uncorrected p-value | FA adjusted p-value | FA F-value | KA Uncorrected p-value | KA adjusted p-value | KA F-value |
| --- | --- | --- | --- | --- | --- | --- |
| Left genu of corpus callosum | 0.898 | 0.927 | 0.108 | 0.678 | 0.981 | 0.395 |
| Right genu of corpus callosum | 0.695 | 0.9 | 0.369 | 0.82 | 0.981 | 0.201 |
| Left body of corpus callosum | 0.708 | 0.9 | 0.35 | 0.928 | 0.981 | 0.075 |
| Right body of corpus callosum | 0.742 | 0.9 | 0.303 | 0.938 | 0.981 | 0.064 |
| Left anterior corona radiata | 0.927 | 0.927 | 0.076 | 0.689 | 0.981 | 0.378 |
| Right anterior corona radiata | 0.802 | 0.909 | 0.223 | 0.473 | 0.981 | 0.772 |
| Left posterior corona radiata | 0.397 | 0.9 | 0.96 | 0.558 | 0.981 | 0.597 |
| Right posterior corona radiata | 0.4 | 0.9 | 0.952 | 0.224 | 0.869 | 1.595 |
| Left superior corona radiata | 0.216 | 0.9 | 1.635 | 0.361 | 0.877 | 1.063 |
| Right superior corona radiata | 0.489 | 0.9 | 0.738 | 0.345 | 0.877 | 1.114 |
| Left posterior thalamic radiation | 0.123 | 0.9 | 2.289 | 0.061 | 0.521 | 3.144 |
| Right posterior thalamic radiation | 0.147 | 0.9 | 2.079 | 0.179 | 0.869 | 1.85 |
| Left Cingulate and Cingulum Gyrus | 0.197 | 0.9 | 1.743 | 0.981 | 0.981 | 0.019 |
| Right Cingulate and Cingulum Gyrus | 0.549 | 0.9 | 0.615 | 0.256 | 0.869 | 1.445 |
| Right superior cerebellar peduncle | 0.585 | 0.9 | 0.548 | 0.833 | 0.981 | 0.184 |
| Right Middle Cerebellar Peduncle | 0.294 | 0.9 | 1.289 | 0.722 | 0.981 | 0.331 |
| Left super longitudinal fascicle | 0.45 | 0.9 | 0.825 | 0.044 | 0.521 | 3.595 |

Table S7: Overview of the uncorrected p-value, corrected p-value, and F-values for MD and MK across each region of interest for the group comparison across American spinal injury association (ASIA) Impairment Scale (AIS) levels in individuals with subacute spinal cord injury.

| ROI | MD Uncorrected p-value | MD adjusted p-value | MD F-value | MK Uncorrected p-value | MK adjusted p-value | MK F-value |
| --- | --- | --- | --- | --- | --- | --- |
| Left genu of corpus callosum | 0.911 | 0.923 | 0.093 | 0.668 | 0.91 | 0.411 |
| Right genu of corpus callosum | 0.762 | 0.923 | 0.275 | 0.744 | 0.91 | 0.299 |
| Left body of corpus callosum | 0.181 | 0.652 | 1.839 | 0.597 | 0.91 | 0.528 |
| Right body of corpus callosum | 0.501 | 0.923 | 0.711 | 0.42 | 0.91 | 0.902 |
| Left anterior corona radiata | 0.851 | 0.923 | 0.163 | 0.65 | 0.91 | 0.439 |
| Right anterior corona radiata | 0.874 | 0.923 | 0.136 | 0.79 | 0.91 | 0.237 |
| Left posterior corona radiata | 0.307 | 0.652 | 1.242 | 0.91 | 0.91 | 0.094 |
| Right posterior corona radiata | 0.277 | 0.652 | 1.356 | 0.73 | 0.91 | 0.319 |
| Left superior corona radiata | 0.121 | 0.652 | 2.309 | 0.769 | 0.91 | 0.265 |
| Right superior corona radiata | 0.661 | 0.923 | 0.421 | 0.295 | 0.91 | 1.286 |
| Left posterior thalamic radiation | 0.01 | 0.168 | 5.633 | 0.297 | 0.91 | 1.277 |
| Right posterior thalamic radiation | 0.203 | 0.652 | 1.705 | 0.725 | 0.91 | 0.325 |
| Left Cingulate and Cingulum Gyrus | 0.041 | 0.345 | 3.674 | 0.068 | 0.91 | 3.02 |
| Right Cingulate and Cingulum Gyrus | 0.659 | 0.923 | 0.425 | 0.201 | 0.91 | 1.716 |
| Right superior cerebellar peduncle | 0.706 | 0.923 | 0.353 | 0.37 | 0.91 | 1.036 |
| Right Middle Cerebellar Peduncle | 0.3 | 0.652 | 1.273 | 0.819 | 0.91 | 0.201 |
| Left super longitudinal fascicle | 0.923 | 0.923 | 0.081 | 0.894 | 0.91 | 0.112 |

Table S8: Clinical correlations with MD. Spearman correlations between MD in the right genu of corpus callosum and clinical variables in individuals with SCI.

| Group | ROI | Metric | Clinical variable | $\rho$ | Unadjusted p-value | Adjusted p-value |
| --- | --- | --- | --- | --- | --- | --- |
| SCI | Right genu of corpus callosum | MD | Total SCIM | -0,510 | 0,006 | **0,022** |
| SCI | Right genu of corpus callosum | MD | Total ASIA pinprick | -0,326 | 0,090 | 0,181 |
| SCI | Right genu of corpus callosum | MD | Total ASIA light touch | -0,267 | 0,170 | 0,227 |
| SCI | Right genu of corpus callosum | MD | Total ASIA motor score | -0,151 | 0,442 | 0,442 |

Table S9: Clinical correlations with MK. Spearman correlations between MK in the right genu of corpus callosum and clinical variables in individuals with SCI.

| Group | ROI | Metric | Clinical variable | $\rho$ | Unadjusted p-value | Adjusted p-value |
| --- | --- | --- | --- | --- | --- | --- |
| SCI | Right genu of corpus callosum | MK | Total SCIM | 0,482 | 0,009 | **0,038** |
| SCI | Right genu of corpus callosum | MK | Total ASIA pinprick | 0,392 | 0,039 | 0,078 |
| SCI | Right genu of corpus callosum | MK | Total ASIA light touch | 0,337 | 0,079 | 0,106 |
| SCI | Right genu of corpus callosum | MK | Total ASIA motor score | 0,276 | 0,156 | 0,156 |

**References**

1. Guo Y, Gao F, Liu Y, Guo H, Yu W, Chen Z, et al. White Matter Microstructure Alterations in Patients With Spinal Cord Injury Assessed by Diffusion Tensor Imaging. Front Hum Neurosci. 2019;13:11.

2. Huynh V, Staempfli P, Luetolf R, Luechinger R, Curt A, Kollias S, et al. Investigation of Cerebral White Matter Changes After Spinal Cord Injury With a Measure of Fiber Density. Front Neurol. 2021;12:598336.

3. Ilvesmaki T, Koskinen E, Brander A, Luoto T, Ohman J, Eskola H. Spinal cord injury induces widespread chronic changes in cerebral white matter. Hum Brain Mapp. 2017;38(7):3637-47.

4. Sun P, Murphy RK, Gamble P, George A, Song SK, Ray WZ. Diffusion Assessment of Cortical Changes, Induced by Traumatic Spinal Cord Injury. Brain Sci. 2017;7(2).

5. Zheng W, Chen Q, Chen X, Wan L, Qin W, Qi Z, et al. Brain White Matter Impairment in Patients with Spinal Cord Injury. Neural Plast. 2017;2017:4671607.

6. Benjamini Y, Hochberg Y. Controlling the False Discovery Rate: A Practical and Powerful Approach to Multiple Testing. Journal of the Royal Statistical Society: Series B (Methodological). 2018;57(1):289-300.
